# Is there a need for anthropometric standards in olympic sports? A proposal for the kinanthropometric profile of elite flag football athletes

**DOI:** 10.3389/fnut.2025.1534453

**Published:** 2025-06-19

**Authors:** Luis Felipe Talavera-Hernández, Claudia Maceroni, Eleanor Louise Travis-Carr, Lewis James Macgregor, Luis Gerardo Vázquez-Villarreal, Rubén Menargues-Ramírez, Martha Patricia Dergal-Irigoyen, Elizabeth Reyes-Castillo, Jesús Iván Castro-Ávila, José Miguel Martínez-Sanz, Nidia Rodriguez-Sanchez

**Affiliations:** ^1^Department of Nutrition, Faculty of Nursing and Nutrition, Autonomous University of Chihuahua, Chihuahua, Mexico; ^2^Sports Science Research, Seattle, WA, United States; ^3^Musculoskeletal Health Research Group, School of Health, Leeds Beckett University, Leeds, United Kingdom; ^4^Faculty of Health Sciences and Sport, University of Stirling, Scotland, United Kingdom; ^5^Medical Direction and Applied Sports Sciences, LFA Professional American Football League, Mexico City, Mexico; ^6^Centro Avantia y Salud, Alicante, Spain; ^7^ABC Medical Center, Mexico City, Mexico; ^8^School of Nutrition, Iberoamerican University, Mexico City, Mexico; ^9^DBSS Science Research, Amerike University, Mexico City, Mexico; ^10^Department of Nursing, Faculty of Health Sciences, Research Group in Applied Dietetics, Nutrition and Body Composition (DANuC), University of Alicante, Alicante, Spain

**Keywords:** flag football, body composition, anthropometry, somatotype, sport performance

## Abstract

**Background:**

Body composition strongly influences the performance of flag football (FF) players, which makes anthropometric measurements important. With the growing popularity of FF, understanding body composition requirements for both male and female players is essential to help optimize their performance. Purpose: This study aimed to characterize and compare anthropometric and body composition profiles between male and female FF players across different playing positions. The study was conducted during the European Flag Football Championship organized by the International Federation of American Football (IFAF).

**Methods:**

A cross-sectional study design was used. Anthropometric measurements followed the International Society for the Advancement of Kinanthropometry (ISAK) full profile protocol. Data were collected from 91 male and 48 female players, and body composition was estimated using the five-way fractionation method. Comparisons between males and females were performed using analysis of variance (ANOVA), with statistical significance set at *p* < 0.05.

**Results:**

Normative reference values and percentiles for anthropometric variables and body composition were established for male and female FF athletes. Male players had an average sum of eight skinfolds of 83.5 ± 30.5 mm, muscle mass of 36.0 ± 7.4 kg, and adipose mass of 19.8 ± 5.1 kg. Female players (27.4 ± 4.5 years) had an average sum of 8 skinfolds of 115.5 ± 40.9 mm, muscle mass of 27.8 ± 4.0 kg, and adipose mass of 20.5 ± 4.8 kg. Both sexes predominantly presented a mesomorphic somatotype.

**Conclusion:**

This study provides valuable data on anthropometric characteristics of male and female FF players. These results can help create normative reference values and support strategies for performance optimization. Additionally, findings contribute to better understanding of body composition needs in FF athletes of both sexes.

## 1 Introduction

Flag football (FF) is a modified form of American tackle football that has gained immense popularity recently, culminating in the International Olympic Committee (IOC) approving inclusion of FF in the Olympic Games program for Los Angeles 2028 (1, 2). With its non-contact nature, the game allows players to enjoy the thrill and excitement of football without the risk of physical harm (3). In FF, players wear flags securely attached to their waists, and the opponents must remove these flags to end a down (4). This rule eliminates the need for tackling and ensures the game remains safe and injury-free (5). Flag football also serves as an excellent platform for developing essential skills required in tackle football. The game’s rules closely resemble the traditional form, allowing players to practice their strategic thinking, agility, and coordination. Participants enhance their ability to execute plays effectively and make split-second decisions with each pass, catch, and maneuvre (6, 7). While much attention is given to the tactical aspects and skill level of players, there is emerging interest in understanding the physiological demands and body composition characteristics of FF athletes. This interest stems from the recognition that a player’s physical attributes, as have been shown in sports in general, can significantly impact their performance and overall success on the field (8, 9).

Comparisons between FF and other sports, such as traditional American football or soccer, reveal both differences and similarities in terms of physiology, tactics, and body composition (BC) (10, 11). Physiologically, FF players prioritize agility, speed, and endurance rather than pure strength and power, as typically seen as desirable for contact sports like American football (12). These differences are deeply related to the BC, adding that FF players also require sufficient strength and explosiveness for rapid change of direction and to evade defenders (13–15).

There is a need for greater understanding of anthropometric characteristics and BC of elite/international level FF players (16), to provide valuable insight into the physiological demands of the sport, and help coaches tailor training programs to enhance performance and minimize injury risk (17, 18). Additionally, we currently lack standardized reference values specific to FF, which could aid talent identification, player development, and performance monitoring, as observed in comparable team sports (19, 20). Furthermore, established anthropometric norms for FF would facilitate comparisons with athletes across different levels in other sports, contributing to deeper understanding of the unique physical demands and adaptations associated with each sport (9, 17, 18).

Sport Science research has extensively explored anthropometric references and BC in team sports such as soccer, basketball, handball (9, 18, 21, 22); however, limited attention has been directed toward understanding the unique physical characteristics of athletes participating in FF. To our knowledge, only two studies to-date have investigated BC profile and anthropometric assessments among FF players (23, 24). These studies determined fat- and fat-free mass via air displacement plethysmography (23), and body fat percentage using the equations described by Jackson and Pollock (1980) and Durnin and Womersley (1974) (24). While these studies provide some valuable insight, collectively only 36 FF players were included, sampled from developmental level female teams. Therefore, if we wish to optimize player development, performance, and injury prevention strategies within this rapidly evolving sport, there is a clear need for empirical investigations, using a standardized approach, to provide essential data specifically addressing the anthropometric measurements and BC profiles of elite/international level FF athletes.

Hence, the aim of this study was to complete a comprehensive analysis of anthropometric measurements and BC profiles of male and female elite/international level FF athletes participating in the 2023 IFAF European Flag Football Championship. We hypothesized differences in height, body mass, skinfold thickness sum, adipose tissue, and muscle mass, based on sex.

## 2 Materials and methods

### 2.1 Design

We conducted a cross-sectional, observational study to determine the anthropometric characteristics, body composition, and somatotypes of elite/international-level FF players sampled from teams competing at the IFAF European Flag Football Championship. The University of Stirling NHS, Invasive or Clinical Research (NICR) Committee granted ethical approval (Reference: NICR 2023 14791 10386). The research design followed the World Medical Association codes and the Helsinki Declaration. In addition, the study design and the development of the manuscript followed the STROBE statement (25).

### 2.2 Participants and sample size

The research population was chosen through non-probabilistic convenience sampling among the 32 international FF teams (19 male teams, 13 female teams) competing in the IFAF European Flag Football Championships in Limerick, Ireland, between 18 and 20 August 2023 (26). To select the sample for this study, we emailed the managing director of IFAF and the coaches of each participating team. They were informed about the study’s characteristics and requested their collaboration. Once they agreed to participate, all players were previously informed of the objectives and method of the research, signing the informed consent before starting the research. The evaluations were conducted during each team’s training schedule. The sample size calculation was performed with Rstudio software (version 3.15.0, Rstudio Inc., Boston, MA, United States). The significance level was set *a priori* at *p* = 0.05. The standard deviation (SD) was to the percentage of adipose tissue (AT) from previous studies (SD = 2.12) (20). With an estimated error (d) of 0.38%. Based on this methodology, the minimum required sample size was 120 participants, with an estimated error (d) of 0.5 for SS percentage within a 95% confidence interval (CI).

A total of 139 FF players, 48 female (mean age: 27.4 ± 4.5 years), 91 male (mean age: 26.6 ± 5.3 years), representing nine European and Western Asia nations (Germany, Great Britain, France, Ireland, Spain, Israel, Georgia, Sweden, and Poland), participated in this study, providing a sample that represented 40.6% of the participant teams (30.7% of females and 47.4% of males). The criteria for inclusion in the study were: (a) be healthy with medical authorization for the practice of federated sport; (b) training a minimum of 5 days per week. The exclusion criteria for the study were: (a) being injured at the time of evaluations, (b) having been injured 1 month before evaluations and (c) having been denied by their team’s head coach to take part in the study. Table 1 describes players’ age, ethnicity, nation and years of experience, segmented by sex.

**TABLE 1:** Descriptive data of the sample by sex in international flag football players (*n* = 139).

		Total (*N* = 139)		Males (*n* = 91, 65.5%)		Females (*n* = 48, 34.5%)	
		Frequency	%	Frequency	%	Frequency	%
Ethnicity	Caucasian	120	86.3	79	86.8	41	85.4
	Mulato	3	2.2	1	1.1	2	4.2
	African	6	4.3	2	2.2	4	8.4
	Middle East	10	7.2	9	9.9	1	2.1
Nation	Great Britain	17	12.2	4	4.4	13	27.1
	Germany	11	7.9	11	12.1	0	0
	Ireland	24	17.3	12	13.2	12	25
	Spain	24	17.3	12	13.2	12	25
	France	23	16.5	12	13.2	11	22.9
	Georgia	6	4.3	6	6.6	0	0
	Israel	10	7.2	10	11	0	0
	Sweden	12	8.6	12	13.2	0	0
	Poland	12	8.6	12	13.2	0	0
Experience (*n* = 108)*	< 3 years	29	26.9	18	24.3	11	32.4
	3–5 years	27	25	21	28.4	6	17.6
	5–7 years	16	14.8	9	12.2	7	20.6
	> 7 years	36	33.3	26	35.1	10	29.4
Player position	Quarterback	15	10.8	11	12.1	4	8.3
	Wide receiver	44	31.7	28	30.8	16	33.3
	Center	15	10.8	10	11	5	10.4
	Defensive back	33	23.7	21	23.1	12	25
	Safety	20	14.4	11	12.1	9	18.8
	Rusher	12	8.6	10	11	2	4.2

*Data from participants who provided this information.

### 2.3 Instruments

Kinanthropometric measurements were performed according to the ISO 7250-1:2017 (27) and the International Society for the Advancement of Kinanthropometry (ISAK) full profile protocol standards for marking and locating (28): four basic measurements, eight skinfold thicknesses, 13 girths, nine lengths and heights, nine breadths and depths. A SECA 862 scale (SECA, Hamburg, Germany) with 100 g accuracy was used to measure body mass; a SECA 217 measuring stadiometer (SECA, Hamburg, Germany) with 1 mm accuracy was used to measure stretch height and sitting height; a wingspan meter (Smartmet, Jalisco, Mexico) with 1 mm accuracy was used to measure arm span; a metallic tape (Lufkin, Mexico) with 1 mm accuracy to measure girths; a small and large sliding caliper (Rosscraft, Canada) with 1 mm accuracy to measure bone breadths, a segmometer (Holway, United States) with 1 mm accuracy to measure length and a Harpenden skinfold caliper (HaB Direct, United Kingdom) of 0.2 mm precision; and finally a wood boxes with a dimension of 30 × 40 × 50 centimeteres (Nutriequipo, Jalisco, Mexico) to help the performance of the evaluation. All kinanthropometric measurements were measured by duplicate or triplicate by an anthropometrist level 2 or 3 accredited by ISAK. The intrer-evaluator TEM was 0.09% for the basic measurements, 2.98% for the skinfolds and 0.88% for the girths and its correlation coefficient with an expert anthropometrist level 4 was 0.99 for the basic measurements, 0.91 for the skinfolds and 0.99 for the girths. All measurements were taken from 09:00 to 19:00 h, depending on when the evaluation of each of the teams included in the study was scheduled based on their availability to take part in the study. Body composition was determined using the equations described by Ross and App (29), Ross and Kerr (30), following the five-component model: adipose mass, muscle mass (MM), bone mass, residual mass, and skin. The sum of six and eight skinfolds and health and proportionality indexes were calculated (31). Somatotype was estimated following the Heath–Carter method, establishing the three Carter components (endomorph, mesomorph, and ectomorph, separately) and representing those results in a somatotype chart. The somatotype chart is the graphical representation of the somatotype where the rating of the three components is plotted in a two-dimensional chart (32, 33). In addition, anthropometric performance indices (Brachial Index (Upper Arm-Forearm Ratio), Crural Index (Thigh-Leg Ratio), Relative Arm Span, Acromio-Iliac Index, Cormic Index, Lower limb relative index, Arm span - height difference, Active body mass index (IAKS), IMLG, Pignet constitutional index, Skeletal index, Chest index (cm), Relative length of upper limb, Relative arm length, Relative forearm length, Relative hand length, Relative length of lower limbs, Relative thigh length, Relative leg length, Relative foot length) and adipose-muscle indexes (Adipose-Muscular Index and Muscle-Bone Index) were calculated (31, 32).

### 2.4 Statistical analysis

Descriptive statistics were expressed as mean ± standard deviation (SD), and frequencies were expressed as percentages. The normality of the transformed data was assessed using the Kolmogorov-Smirnov test, alongside the examination of skewness and kurtosis. The results indicated that the transformed data followed a normal distribution, thus permitting the application of parametric tests for the analysis. Differences between continuous variables were assessed using an independent Student’s *t*-test when compared by sex, the data were transformed using the base 10 logarithm to use parametric tests. Percentiles were reported at every 10 points of the scale relating to all the variables included in the analysis, considering what has been done in similar research in team sports (20). Somatotype attitudinal distance (SAD) was reported to evaluate the distance in three dimensions between two somatotype points, for this case winners somatotype were used as reference, somatotype attitudinal mean (SAM) was also reported by sex (33). We used the IBM SPSS Statistics program (v.25; IBM Inc, Chicago, IL, United States) for all data analyses, with significance set at *p* < 0.05.

Information on the anthropometric profile and percentiles by sex can be found in the Table 5.

## 3 Results

The anthropometric variables and body composition report statistical differences between sex and playing positions at the highest level of competition in football players. As shown in Table 2, male athletes were on average 15 cm taller and almost 17 kg heavier than female athletes (*p* < 0.001), but the main difference in mass was due to bone and muscle mass, since there was no significant difference in adipose mass (*p* = 0.275), although this average corresponds to more than 30% of women and just 23% of men (*p* < 0.001). It should be noted that, although there were no differences in adipose mass between the two sexes, differences of ∼30 mm in the thickness sums of six and eight skinfolds were observed, with smaller skin fold thickness among men compared to women (*p* < 0.001). On the other hand, male players’ bone mass was up to 2 kg greater than female players’ (*p* < 0.001), but if we consider the percentage that bone mass represents in body composition, no difference was observed (11.4%, *p* = 0.553).

**TABLE 2:** Anthropometric and body composition variables by sex in international flag football players (*n* = 139), presented as means and standard deviations (SD).

	Total (*N* = 139)		Males (*n* = 91)		Females (*n* = 48)		*P*
	Mean	SD	Mean	SD	Mean	SD	
Body mass (kg)	77.3	12.9	83.1	10.9	66.3	8.6	< 0.001**
Stretch stature (cm)	176.1	9.9	181.5	7.1	165.8	5.2	< 0.001**
Sitting height (cm)	92.8	4.8	95.3	3.6	88.2	3.1	< 0.001**
Arm span (cm)	178.5	11.2	184.8	7.7	166.7	6.0	< 0.001**
Triceps SF (mm)	11.8	5.8	9.5	4.6	16.3	5.2	< 0.001**
Subscapular SF (mm)	10.8	4.3	10.2	3.5	12.0	5.2	0.019*
Biceps SF (mm)	4.9	3.2	3.8	1.6	7.0	4.2	< 0.001**
Iliac crest SF (mm)	14.9	6.7	14.5	6.1	15.7	7.8	0.322
Supraspinale SF (mm)	9.4	5.2	8.7	4.1	10.8	6.7	0.025*
Abdominal SF (mm)	16.3	7.1	16.3	6.7	16.4	8.0	0.885
Thigh SF (mm)	16.1	8.3	12.6	6.1	22.7	7.8	< 0.001**
Calf SF (mm)	10.2	5.8	7.9	4.0	14.6	6.2	< 0.001**
Head girth (cm)	56.3	1.8	56.9	1.6	55.2	1.7	< 0.001**
Neck girth (cm)	36.4	3.4	38.4	1.8	32.6	2.2	< 0.001**
Arm relaxed girth (cm)	32.0	3.1	33.4	2.3	29.3	2.6	< 0.001**
Arm flexed and tensed girth (cm)	33.4	3.5	35.3	2.3	29.8	2.3	< 0.001**
Forearm girth (cm)	27.4	2.4	28.8	1.3	24.9	1.6	< 0.001**
Wrist girth (cm)	16.4	1.3	17.2	0.9	15.1	0.7	< 0.001**
Chest girth (cm)	98.1	7.8	102.0	5.8	90.7	5.1	< 0.001**
Waist girth (cm)	80.5	7.7	83.8	6.0	74.2	6.3	< 0.001**
Hips girth (cm)	100.8	6.3	101.3	6.2	99.9	6.4	0.198
Thigh 1 cm gluteal girth (cm)	61.2	4.2	61.5	4.0	60.7	4.4	0.284
Thigh middle girth (cm)	55.6	3.9	56.3	3.5	54.1	4.1	0.001*
Calf girth (cm)	38.0	2.4	38.6	2.4	37.0	2.0	< 0.001**
Ankle girth (cm)	22.8	1.5	23.2	1.3	21.8	1.5	< 0.001**
Acromiale-Radiale length (cm)	33.9	2.2	34.9	1.7	31.9	1.7	< 0.001**
Radiale-Stylion length (cm)	26.0	1.8	26.9	1.4	24.3	1.3	< 0.001**
Midstylion-Dactylion length (cm)	19.5	1.6	20.1	1.2	18.2	1.4	< 0.001**
Iliospinale height (cm)	98.5	6.5	101.8	5.1	92.2	3.8	< 0.001**
Trochanterion height (cm)	92.1	6.5	95.1	5.6	86.4	3.9	< 0.001**
Trochanterion-Tibiale laterale length (cm)	45.2	5.4	45.9	3.7	44.0	7.5	0.042*
Tibiale Laterale height (cm)	47.3	3.8	49.2	2.9	43.6	2.3	< 0.001**
Foot length (cm)	26.1	1.8	27.0	1.3	24.3	1.1	< 0.001**
Tibiale Mediale-Sphyrion Tibiale length (cm)	39.4	2.8	40.7	2.4	37.0	1.8	< 0.001**
Biacromial breadth (cm)	40.5	3.0	42.1	2.3	37.6	1.6	< 0.001**
Biiliocristal breadth (cm)	28.9	2.4	29.4	2.5	28.1	2.0	0.002*
Transverse chest breadth (cm)	29.2	2.6	30.3	2.5	27.1	1.4	< 0.001**
A-P chest depth (cm)	19.0	2.3	19.9	1.9	17.3	1.9	< 0.001**
Humerus breadth (cm)	7.0	0.6	7.3	0.4	6.4	0.3	< 0.001**
Bi-Styloid breadth (cm)	5.7	0.5	5.9	0.4	5.2	0.3	< 0.001**
Femur breadth (cm)	9.8	0.7	10.1	0.6	9.3	0.5	< 0.001**
Bimalleolar breadth (cm)	7.3	0.6	7.6	0.5	6.6	0.3	< 0.001**
Muscle mass (kg)	36.0	7.4	40.4	4.6	27.8	4.0	< 0.001**
Adipose mass (kg)	19.8	5.1	19.5	5.2	20.5	4.8	0.275
Bone mass (kg)	8.8	1.7	9.5	1.5	7.5	1.0	< 0.001**
Skin mass (kg)	3.9	0.5	4.1	0.5	3.7	0.3	< 0.001**
Residual mass (kg)	8.7	1.9	9.6	1.5	6.8	1.1	< 0.001**
% of Muscle mass	46.5	4.9	48.8	3.7	42.1	3.9	< 0.001**
% of Adipose mass	25.8	5.5	23.2	3.8	30.7	4.7	< 0.001**
% of Bone mass	11.4	1.0	11.5	0.9	11.3	1.2	0.553
% of Skin mass	5.2	0.7	5.0	0.6	5.6	0.5	< 0.001**
% of Residual mass	11.1	1.0	11.6	0.8	10.3	0.7	< 0.001**
Summation of 8 skinfolds (mm)	94.6	37.5	83.5	30.5	115.5	40.9	< 0.001**
Summation of 6 skinfolds (mm)	74.7	30.4	65.2	24.8	92.8	32.0	< 0.001**

*Significative differences in *t*-student test *p* < 0.05. **Significant differences in *t*-student test *p* ≤ 0.01.

In terms of the distribution of adipose tissue in different regions of the body, differences in the upper and lower extremities were observed, where female athletes averaged 26.8 mm greater mass in triceps, biceps, thigh, and calf skinfolds (*p* < 0.001). We can also observe that, as expected, girths were greater in males in comparison to females because of the differences in body mass and height. Similar cases occurred with bone lengths and diameters, most of which were reported to be greater in men than in women (*p* < 0.001).

**FIGURE 1:**
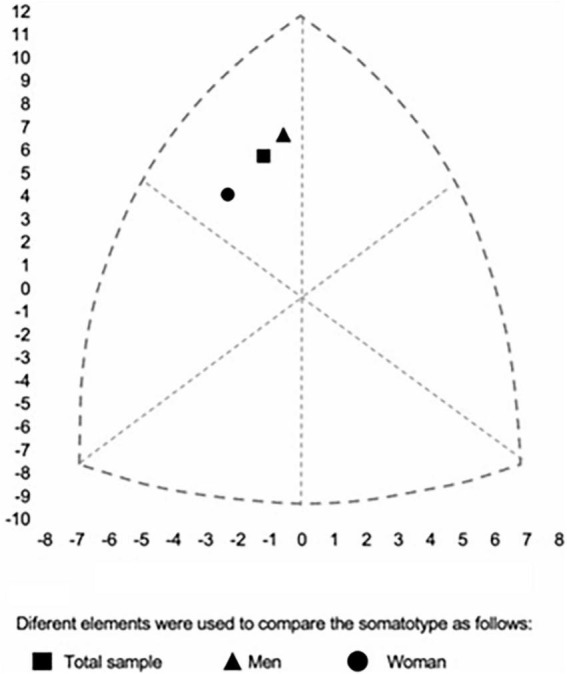
Somatotype of flag fotball players by total sample and sex. Diferent elements were used to compare the somatotype as follows 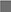 Total sample, 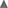 Men, 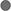 Woman.

In addition to body composition, Table 3 shows the results of the estimation of body shape with somatotype, which consists of three dimensions: adipose tissue deposit (endomorphy), development of muscle and skeletal tissue (mesomorphy), and linearity (ectomorphy). These data can also be seen in Figure 1, in which we observe that the main component in the population is the mesomorphy, placing both men and women in the category of mesomorph - endomorphic, although technically belonging to the same category, males and females are at the extreme of it (*p* < 0.001), with a significant development of mesomorphy over the other components in males, and no differences between mesomorphy and endomorphy in females.

**TABLE 3:** Somatotype components by sex in international flag football players (*n* = 139), presented as means and standard deviations (SD).

	Total (*N* = 139)		Males (*n* = 91)		Females (*n* = 48)		*P*
	Mean	SD	Mean	SD	Mean	SD	
Endomorphy	3.1	1.3	2.6	1.0	4.0	1.4	< 0.001**
Mesomorphy	5.3	1.1	5.6	1.0	4.8	1.1	< 0.001**
Ectomorphy	1.9	1.0	2.0	0.9	1.7	1.1	0.050
X	−1.2	2.1	−0.6	1.7	−2.3	2.2	< 0.001**
Y	5.7	2.8	6.6	2.5	4.0	2.5	< 0.001**

*Significative differences in *t*-student test *p* < 0.05. **Significant differences in *t*-student test *p* < 0.01.

Various indices that may be related to sports performance and the incidence of injuries have been estimated from anthropometric variables (Table 4). The adipose-muscle and bone-muscle indices relativize proportions of different tissues of the body, better showing the level of efficient tissues relative to another, helping to define the specific range that FF players should consider. The muscle adiposity index indicates the amount of fat tissue per kilogram of muscle mass, with males exhibited about 250 g/kg less than females (*p* < 0.001), but at the same time, males presented 600 g of muscle mass per kilogram of bone mass more than females.

**TABLE 4:** Anthropometric performance indices and adipose-muscle indices by sex in international flag football players (*n* = 139), presented as means and standard deviations (SD).

	Total (*N* = 139)		Males (*n* = 91)		Females (*n* = 48)		*P*
	Mean	SD	Mean	SD	Mean	SD	
Adipose-Muscular index	575.3	192.2	484.7	122.4	746.9	183.8	< 0.001**
Muscle-Bone index (kg)	4.1	0.6	4.3	0.5	3.7	0.5	< 0.001**
Brachial index (upper arm-forearm ratio) (cm)	76.9	3.5	77.2	3.3	76.2	3.9	0.092
Crural index (thigh-leg ratio) (cm)	87.8	8.0	89.1	7.2	85.4	8.8	0.009*
Relative arm span (cm)	101.4	2.0	101.8	1.8	100.5	2.3	< 0.001**
Acromio-Iliac index (cm)	71.6	5.0	69.9	4.4	74.8	4.7	< 0.001**
Cormic index (trunk-height) (cm)	52.7	1.2	52.5	1.2	53.2	1.2	0.002*
Lower limb relative index (cm)	89.7	4.4	90.6	4.2	88.1	4.3	0.002*
Arm span - height difference (cm)	2.4	3.5	3.2	3.2	0.8	3.7	< 0.001**
% of arm span - height difference	98.7	2.0	98.3	1.7	99.5	2.3	0.001*
Active body mass index (IAKS) (gr/cm^3^)	1.051	0.188	1.144	0.114	0.876	0.177	< 0.001**
IMLG (kg/m^2^)	0.0019	0.0004	0.0021	0.0002	0.0015	0.0003	< 0.001**
Pignet constitutional index	0.7	13.7	−3.6	12.3	8.9	12.5	< 0.001**
Skeletal index (cm)	89.7	4.4	90.6	4.2	88.1	4.3	0.002*
Chest index (cm)	155.5	21.2	153.8	23.8	158.5	14.7	0.216
Relative length of upper limb (cm)	45.0	1.1	45.2	1.0	44.8	1.4	0.075
Relative arm length (cm)	19.2	0.7	19.2	0.6	19.2	0.8	0.834
Relative forearm length (cm)	14.8	0.5	14.8	0.5	14.6	0.6	0.017*
Relative hand length (cm)	11.0	0.6	11.1	0.6	11.0	0.7	0.317
Relative length of lower limbs (cm)	52.3	2.0	52.4	2.1	52.1	2.0	0.468
Relative thigh length (cm)	25.7	3.0	25.3	1.8	26.5	4.5	0.022*
Relative leg length (cm)	26.8	1.1	27.1	1.1	26.3	1.0	< 0.001**
Relative foot length (cm)	14.8	0.5	14.9	0.5	14.7	0.5	0.028*

*Significative differences in *t*-student test *p* < 0.05. **Significant differences in *t*-student test *p* < 0.01.

Other indices refer to the length or width of bones that perform important functions in movement dynamics and can therefore be subsequently associated with sports performance and the incidence of injuries, such as the brachial, crural, and acromio-iliac indices. Brachial index, which distinguishes between arm length and forearm length, there were no differences between sexes (*p* = 0.092), and the length of both bones was relatively similar; this was not the case with crural index, where males had an average length of legs longer than the thigh, while females had a proportional length between the two sections of their legs (*p* = 0.009). In addition, acromio-iliac index, known as the relationship between the width of the biacromial and iliac crests, shows differences statistically significant by sex (*p* < 0.001), although both categorically belong to the upper limit that defines the trunk as trapezoid.

Bone dimensions were also relativized to height in order to be able to analyze different body segments, of which we can highlight that for both sexes, relative arm span is categorized as medium due to the proportional length between arms and height, the above despite their statistical differences (*p* < 0.001), cormic and skeletal indices were similar cases that categorizes both sexes with a medium trunk size and legs proportional to the trunk size, respectively, also happened for the length of feet and hands that were reported in medium size categories.

Finally, Table 5 shows the percentile distribution of the different anthropometric variables and body composition divided by sex.

**TABLE 5:** Percentile distribution of anthropometric variables by sex in international flag football players (*n* = 139).

	Males (*n* = 91)										Females (*n* = 48)									
	10	20	30	40	50	60	70	80	90	100	10	20	30	40	50	60	70	80	90	100
**Basics measurements**																				
Body mass (kg)	71.1	76.1	77.7	79.9	82.0	83.7	86.6	88.4	100.9	123.6	55.0	59.8	62.4	64.1	65.9	66.9	71.0	72.7	77.0	93.9
Stretch stature (cm)	172.5	175.4	178.6	179.9	181.0	183.0	184.4	186.9	190.6	202.7	158.7	161.7	163.0	164.1	165.0	166.6	168.2	171.0	172.6	179.4
Sitting height (cm)	90.7	92.4	93.2	94.5	95.6	96.4	97.3	98.2	100.1	102.7	84.0	85.2	86.6	87.4	88.0	88.7	89.6	90.5	92.5	96.0
Arm span (cm)	174.5	178.5	181.8	183.5	184.2	186.8	188.1	190.4	194.2	206.6	158.5	161.0	164.3	165.6	166.1	167.9	170.4	171.9	173.3	180.3
**Skinfolds measurements**																				
Triceps SF (mm)	4.8	5.6	6.8	7.6	8.2	9.0	11.1	12.7	15.9	30.0	10.8	11.9	13.1	14.0	15.1	16.1	19.1	20.5	24.2	28.4
Subscapular SF (mm)	7.0	7.3	8.2	8.8	9.2	10.2	11.1	12.4	14.6	28.0	6.8	7.9	8.5	10.0	11.2	12.2	12.9	15.2	20.1	31.8
Biceps SF (mm)	2.4	2.8	3.0	3.2	3.4	3.6	4.0	4.5	5.9	13.0	3.0	3.6	4.0	4.5	5.5	7.4	8.3	10.1	15.6	18.2
Iliac crest SF (mm)	7.8	9.2	10.2	12.2	13.0	14.9	17.2	20.0	22.9	34.0	7.0	9.4	10.2	12.4	14.1	16.0	18.6	22.6	25.5	39.0
Supraspinale SF (mm)	5.0	5.8	6.3	6.8	7.4	8.4	9.7	11.2	13.2	24.6	4.4	5.9	6.6	7.8	8.6	10.9	11.4	13.4	23.1	35.0
Abdominal SF (mm)	9.0	10.1	11.5	13.4	15.2	16.8	19.8	21.0	25.8	42.0	7.8	9.6	11.4	12.0	13.5	16.2	20.0	24.4	29.7	38.0
Thigh SF (mm)	5.9	8.1	9.2	10.2	11.0	12.8	14.2	16.3	20.2	39.0	14.1	16.8	18.0	19.2	21.1	23.8	25.7	27.8	31.3	50.5
Calf SF (mm)	4.0	4.9	5.4	6.2	7.0	7.8	8.3	10.1	13.2	24.0	7.0	9.8	10.8	12.8	13.2	14.9	17.1	19.7	23.9	31.0
**Girths measurements**																				
Head girth (cm)	54.5	55.5	56.0	56.7	57.0	57.3	57.7	58.2	58.8	60.4	53.0	53.3	54.1	54.8	55.5	55.8	56.4	56.5	57.2	59.0
Neck girth (cm)	35.8	37.0	37.5	38.0	38.5	39.0	39.5	40.0	40.7	43.6	30.3	30.5	31.1	32.0	32.3	32.8	33.4	34.8	36.2	37.6
Arm relaxed girth (cm)	30.5	31.4	32.1	32.7	33.2	33.8	34.5	35.2	36.9	41.6	25.8	27.2	28.1	28.7	29.5	29.9	30.5	31.0	32.8	36.5
Arm flexed and tensed girth (cm)	32.4	33.2	34.1	34.5	35.2	35.7	36.7	37.3	38.5	41.7	26.7	27.9	28.9	29.6	30.1	30.3	30.7	31.4	32.7	37.0
Forearm girth (cm)	27.0	27.7	28.1	28.4	28.7	29.1	29.4	30.2	30.6	32.3	23.0	23.5	24.2	24.4	24.6	25.0	25.4	25.7	27.5	29.3
Wrist girth (cm)	16.0	16.5	16.7	17.0	17.1	17.3	17.5	18.0	18.5	20.0	14.3	14.5	14.6	15.0	15.0	15.1	15.3	15.6	16.0	17.0
Chest girth (cm)	95.0	97.9	99.4	100.5	101.6	103.3	104.2	106.7	109.0	121.0	85.3	87.4	88.0	89.3	91.1	92.3	92.8	93.3	95.7	104.3
Waist girth (cm)	77.2	78.6	80.0	81.5	84.0	84.6	86.3	88.2	92.4	105.0	67.3	69.4	70.7	72.2	73.7	75.0	76.9	78.4	81.6	93.0
Hips girth (cm)	94.3	96.3	98.1	99.2	100.5	101.3	103.6	105.8	111.6	123.9	89.5	94.4	96.7	99.0	100.0	101.1	102.7	105.3	108.6	113.5
Thigh 1 cm gluteal girth (cm)	56.7	58.0	59.3	60.2	61.5	62.2	63.0	64.5	66.3	75.3	55.3	57.0	58.1	59.2	61.0	62.0	63.1	64.6	66.4	70.0
Thigh middle girth (cm)	51.6	53.9	54.6	55.5	56.0	57.0	57.6	58.8	61.7	67.4	47.0	51.2	52.4	53.7	54.5	55.2	56.2	56.8	59.1	62.9
Calf girth (cm)	35.7	36.3	37.5	38.1	38.6	39.2	39.6	40.1	42.1	44.9	34.3	35.7	36.2	36.5	37.1	37.5	38.1	38.4	39.1	42.7
Ankle girth (cm)	21.5	22.0	22.5	22.8	23.1	23.5	23.8	24.1	25.0	27.9	20.0	20.5	20.9	21.3	21.8	22.0	22.4	23.0	24.0	27.1
**Length and height measurements**																				
Acromiale-Radiale length (cm)	32.8	33.4	34.0	34.4	35.0	35.4	35.8	36.2	37.0	40.1	29.7	30.3	30.8	31.4	32.0	32.3	32.4	33.3	34.4	35.8
Radiale-Stylion length (cm)	25.0	25.8	26.2	26.6	27.0	27.2	27.6	28.0	28.7	30.6	22.8	23.1	23.6	23.8	24.2	24.5	25.0	25.7	26.1	26.6
Midstylion-Dactylion length (cm)	18.6	19.0	19.5	19.9	20.3	20.6	20.9	21.1	21.3	23.0	16.8	17.6	17.8	18.2	18.3	18.7	19.0	19.4	19.7	20.1
Iliospinale height (cm)	95.1	97.1	99.2	100.4	101.8	102.6	103.9	105.4	109.2	117.2	87.6	88.9	90.1	90.8	92.4	93.1	93.9	95.2	97.5	101.5
Trochanterion height (cm)	88.8	90.4	92.1	93.4	94.2	96.5	98.0	100.1	101.4	109.9	81.7	83.1	84.2	85.0	85.9	87.6	88.6	89.7	90.2	99.7
Trochanterion-Tibiale laterale length (cm)	41.2	42.7	44.0	45.1	46.3	46.7	47.7	48.9	50.3	55.5	38.7	40.7	41.2	42.1	42.5	43.7	44.5	45.0	49.1	89.8
Tibiale Laterale Height (cm)	45.4	47.1	48.0	48.4	48.9	49.3	50.0	51.4	53.8	56.5	40.7	41.7	42.1	42.8	43.8	44.1	44.4	45.3	46.7	50.4
Foot length (cm)	25.1	25.9	26.3	26.6	27.0	27.2	27.7	28.1	28.9	31.3	23.0	23.2	23.5	24.1	24.4	24.8	25.2	25.4	25.6	26.2
Tibiale Mediale-Sphyrion Tibiale length (cm)	37.6	38.5	39.6	40.2	40.6	41.3	41.5	42.6	43.8	47.8	34.2	35.2	36.1	36.5	37.1	37.6	38.0	38.2	39.4	41.0
**Breadths measurements**																				
Biacromial breadth (cm)	39.5	40.3	41.0	41.4	42.0	42.5	43.0	44.0	44.6	48.9	35.3	35.9	36.7	37.2	37.6	37.9	38.5	39.0	40.0	41.0
Biiliocristal breadth (cm)	26.6	27.8	28.2	28.4	28.8	29.4	30.0	31.0	32.9	39.6	25.5	26.6	27.0	27.4	27.7	28.1	29.0	29.6	30.6	33.1
Transverse chest breadth (cm)	27.5	28.5	29.0	29.5	30.0	30.5	31.3	32.4	33.1	42.7	25.6	25.9	26.4	26.7	27.3	27.5	27.7	28.0	28.6	32.2
A-P chest depth (cm)	17.5	18.6	19.2	19.6	20.0	20.3	20.7	21.4	22.1	24.4	15.2	15.7	16.1	16.5	16.8	17.3	18.0	18.8	20.3	22.0
Humerus breadth (cm)	6.8	7.0	7.2	7.2	7.3	7.4	7.5	7.7	7.8	8.5	5.9	6.1	6.2	6.2	6.4	6.5	6.5	6.6	6.8	7.1
Bi-Styloid breadth (cm)	5.5	5.6	5.7	5.8	5.9	6.0	6.1	6.3	6.4	7.0	4.7	5.0	5.0	5.1	5.2	5.2	5.3	5.4	5.6	5.8
Femur breadth (cm)	9.5	9.6	9.7	9.9	10.0	10.2	10.3	10.5	10.8	12.1	8.6	8.8	9.0	9.1	9.2	9.4	9.6	9.7	9.9	10.9
Bimalleolar breadth (cm)	7.1	7.3	7.4	7.5	7.6	7.8	7.9	8.0	8.3	8.7	6.2	6.3	6.4	6.5	6.7	6.7	6.8	6.9	7.0	7.4
**Body composition**																				
Muscle mass (kg)	33.8	36.4	38.2	40.0	40.7	41.3	42.4	44.0	45.7	52.9	21.9	24.6	26.8	27.7	28.0	29.7	30.1	31.1	31.9	36.7
Adipose mass (kg)	14.1	16.1	16.8	17.4	18.4	19.3	20.6	23.0	25.2	42.6	14.8	16.4	17.6	18.4	20.4	21.1	21.7	24.5	29.1	33.5
Bone mass (kg)	7.6	8.4	8.7	9.1	9.4	9.8	10.0	10.4	11.5	14.9	6.3	6.6	6.9	7.3	7.4	7.7	7.9	8.0	8.8	10.3
Skin mass (kg)	3.7	3.9	4.0	4.0	4.1	4.2	4.2	4.3	4.5	5.0	3.3	3.5	3.6	3.6	3.7	3.7	3.8	3.9	4.1	4.3
Residual mass (kg)	7.9	8.5	8.8	9.3	9.5	9.8	10.1	10.6	11.9	14.6	5.2	6.1	6.2	6.4	7.0	7.1	7.3	7.6	8.4	9.7
Body mass (kg)	67.2	73.4	76.4	79.8	82.1	84.4	87.4	92.3	98.8	130.0	51.5	57.2	61.1	63.4	66.4	69.4	70.8	75.2	82.3	94.5

## 4 Discussion

This study presents and analyses the anthropometric and performance variables of flag football players from 13 national teams (nine male and four female) who competed at the IFAF 2023 Flag Football European Championships. The aim was to establish normative data for FF players and explore potential associations between anthropometric variables. Through this research, we intend to provide valuable insights that may support the development and advancement of flag football as a competitive sport at various levels, including future inclusion in the Olympic Games.

Sport science research has extensively explored anthropometric references and body composition in team sports such as soccer, basketball, and handball (9, 18, 21, 22), with limited attention has been directed toward understanding the unique physical characteristics of athletes participating in FF. To our knowledge, only two studies to date have investigated body composition profiles and anthropometric assessments among FF players (23, 24). These studies determined adipose mass and fat-free mass via air displacement plethysmography (23) and body fat percentage using equations described by Jackson and Pollock (1978) and Durnin and Womersley (1974) (24). While these studies provide valuable insights, they collectively included only 36 FF players, sampled from developmental-level female teams.

Comparisons between FF and other sports, such as American or European football, highlight notable differences in physiological demands and, consequently, optimal body composition (9, 19). At present, very few studies have looked at the anthropometric profiles of FF athletes. Sebastiá-Rico et al. recently published two studies on the assessment of body composition in professional male soccer players, where the differences between different measurement methods and different playing positions were analyzed (9, 19). The anthropometric profile of Spanish soccer players in different competitive categories has also been published (20). It is very interesting to replicate this type of analysis in FF to establish the reference anthropometric profile in this sport. In individual sports like track and field, sprinters tend to have greater lean muscle mass and lower body fat percentages compared to endurance runners, illustrating how body composition adapts to the specific demands of each discipline (32, 34). In FF, these differences may also be important, adding that players also require sufficient strength and explosiveness for a rapid change of direction and to evade defenders (14, 15).

Alves Junior et al. (23) published a paper exploring the relationship between body composition and athletic performance of female athletes across three different sports (flag football, *n* = 12; indoor soccer, *n* = 20; volleyball, *n* = 13) utilizing air displacement plethysmography and dual-energy X-ray absorptiometry to measure body adipose mass and fat-free mass (23). The results compared similarly to our findings of the height of 165.15 cm ± 5.06 (offense: 165.0 cm ± 5.1, defense; 167.0 cm ± 5.3) but a fat mass of 16.00 kg ± 1.70 using the Siri equation (35) which was greater (offence: 21.01 kg ± 4.85; defense; 20.49 kg ± 5.17) than through Kerr’s equation (29). However, it’s important to mention that the technique and equation differed in this study compared to ours. To accurately compare our findings, it is crucial to employ the same anthropometric method and equations to draw accurate conclusions. Because of this, it is important to note that the current methodologies for estimating different body composition components vary depending on the equations or fractionation models selected by researchers (9, 19, 20). Often, authors choose equations that have not been validated for athletic populations or confuse terms related to lipid mass, fat mass, and adipose tissue mass, as well as the two, four and five-component fractionation models (9, 19, 32). Currently, the most precise and accurate method for body mass fractionation is the five-component model proposed by Kerr and App (29), Ross and Kerr (30). Despite the lack of anthropometric data on flag football, there is a multitude of studies on other ball-handling team sports. Bosch et al. (36) found that in American football, flag football’s predecessor, linemen generally had increased adipose mass and lean mass compared to other positions, likely due to the greater physical demands and frequent contact of their role, requiring more muscle and overall mass (36). This agrees with our finding that muscle mass (in kg) was greater in female defensive roles compared to offensive. However, due to the non-contact nature of the sport, some positions might be less specialized in flag football compared to American football, and players may alternate between roles more fluidly (12, 14). Due to a lack of studies into female American football players, this finding has no support in the literature. In addition, no such differences were found in this study regarding muscle mass or any other attributes in men.

The evaluation of anthropometric variables and body composition of male and female FF players reveals significant differences between sexes, which underlines the importance of considering gender when analyzing physical characteristics and training needs in this sport. Male players have significantly higher values for body mass, height, absolute and percentage muscle mass, as well as several measures of body perimeters and lengths, compared to their female counterparts (32, 37). In contrast, female players show higher values for skinfolds and percentage of adipose mass. These differences may influence performance and gender-specific training strategies. For example, the higher muscle mass and lower fat percentage in men may be associated with greater power and speed (14, 38), whereas women, having a higher proportion of fat mass, may benefit from training program focused on reducing fat and increasing lean muscle mass. Furthermore, differences in measures such as arm span and limb length suggest that positions and roles within the team could be allocated with these specific physical characteristics in mind (34, 37).

Bone mass adapts to repetitive impact and mechanical loading, promoting increased bone density. Athletes in high-impact sports such as volleyball, basketball, and gymnastics typically exhibit greater bone parameter values than those with lower mechanical impact, like softball and swimming (39–41). When analyzing bone differences between males and females after adjusting for height, although statistically significant differences are found (*p* < 0.05), categorically and biologically no relevant differences between the sexes are observed. It is advisable to consider relative dimensions as a means of defining a profile of flag football players.

Moreover, the sum of eight skinfolds is commonly used to estimate body fat or adipose mass. Taking the sum of the triceps, subscapular, biceps, iliac crest, supraspinale, abdominal, thigh and calf skinfolds, the method is widely used in sports and health assessments to help understand an individual’s body composition, particularly the proportion of fat relative to lean mass (18–20). The adipose-muscular index, derived using Kerr’s equation, helps assess physique makeup and monitor training and nutritional outcomes. Huovinen et al. (42) noted that individuals with a body fat percentage over 10% showed greater improvements in explosive power, as measured by vertical jumps, with increases ranging from 6 to 14% (42). Abidin and Adam (43) support this research, showing that leaner bodies were linked to better performances in the jump tests, finding that vertical jump height of martial arts athletes can be predicted by the percentage of body fat, emphasizing reducing body fat in lower body power expression (43).

Comparison of somatotypes and performance indices between male and female flag football players reveals significant differences in several anthropometric and body composition dimensions. Males show greater mesomorphy (5.6 ± 1.0 vs. 4.8 ± 1.1, *p* < 0.001) and ectomorphy (2.0 ± 0.9 vs. 1.7 ± 1.1, *p* = 0.050), while females show greater endomorphy (4.0 ± 1.4 vs. 2.6 ± 1.0, *p* < 0.001). These differences in somatotype suggest variations in the distribution of muscle and adipose mass between the sexes, which could influence the physical and tactical demands of the game (32, 37, 38, 44). However, it is important to acknowledge that, at present, there is limited evidence regarding whether somatotype is directly related to body composition or performance outcomes in flag football. In addition, due to the nature of the current dataset, we were unable to evaluate individual or team performance at the elite level in relation to somatotype. Further research is needed to explore and confirm these potential associations.

In terms of anthropometric indices, males have significantly higher values for muscle-bone index (4.3 ± 0.5 vs. 3.7 ± 0.5, *p* < 0.001) and active body mass index (1.1 ± 0.1 vs. 0.9 ± 0.2, *p* < 0.001), suggesting a higher proportion of muscle mass and lower proportion of adipose mass compared to females. On the other hand, females have a significantly higher adipose-to-muscle ratio (746.9 ± 183.8 vs. 484.7 ± 122.4, *p* < 0.001), indicating a greater accumulation of adipose tissue relative to muscle mass (32, 37, 38, 44).

These differences may have direct implications for performance and training strategy. Men, with greater muscle mass and lower adiposity, may be better prepared to perform in positions requiring strength and explosive speed, while women may benefit from training programs focused on increasing lean muscle mass and reducing adipose mass to improve performance (44). Furthermore, differences in indices such as crural (89.1 ± 7.2 vs. 85.4 ± 8.8, *p* = 0.009) and relative arm span (101.8 ± 1.8 vs. 100.5 ± 2.3, *p* < 0.001) highlight the need to tailor playing tactics and positional allocation according to the specific physical characteristics of each player (32, 37).

The distinctions between contact and non-contact sports elicit major modifications in gameplay strategy. Flag football emphasizes strategic plays focusing on speed, agility, and tactical execution without the need for peak strength and greater mass required in physical contact, more akin to basketball or soccer. Instead, in similar ball-handling team sports like flag football, the power-to-weight ratio is shown to be a predictor of competitive success, especially in sports where agility and quickness and an element of jumping are paramount (45, 46). In contrast to some sports where body composition significantly correlates with athletic skills, different studies found no significant correlation between morphological variables and soccer-specific technical skills, suggesting that body composition and anthropometric attributes are not reliable predictors of soccer skills in female players (47, 48). Conversely, Čavala et al. (49) found that explosive strength and greater muscle mass significantly contributed to the quality of handball performance. Our findings that leaner and more muscularly developed bodies were associated with better performances in the jump tests highlight the importance of body composition’s role in lower body power expression.

Overall, these findings highlight the importance of considering gender differences in the assessment and training planning of flag football players, allowing for a more personalized and effective approach that maximizes performance and minimizes the risk of injury (5, 14, 20, 37).

### 4.1 Application percentiles

Due to the variability that exists among body dimensions in the population due to factors such as age, gender, ethnicity, physical exercise, and diet (32, 37), percentiles can serve as a valuable statistical tool for assessing and comparing anthropometric, body composition, and somatotype measurements in a specific population (32, 50). Percentiles represent the value below which a certain percentage of the population falls for a given measure, with the 50th percentile being the median, dividing the population into two equal parts and serving as a reference point for the population under evaluation (20). These can be applied to evaluate physical performance or monitor the body composition of a soccer player or group of players against the reference data we provide from Spanish soccer. This tool does not offer information on health or sports performance, so its interpretation should be done cautiously depending on the anthropometric variable being compared (9, 19). FM estimation with different formulas cannot be compared (9, 19, 50), making it necessary to have a percentile table, as included in the current study, to determine equivalences between methods.

In summary, coaching and medical staff can use percentiles to evaluate a player’s individual progress over time. For instance, if a player is at the 50th percentile for MM and, after a period of training, their position moves to the 90th percentile, this indicates a significant improvement in their MM, allowing the coaching staff to tailor training programs more precisely.

### 4.2 Future research

A greater understanding of the anthropometric characteristics and body composition of elite and international-level flag football (FF) players is needed (16) to provide valuable insight into the physiological demands of the sport and to assist coaches in designing training programs aimed at enhancing performance and reducing injury risk (17, 18). Investigating how body composition changes throughout the season —from the pre-season, which focuses on increasing muscle mass and reducing adipose mass, to the competitive season, where the emphasis is on maintaining fitness and optimizing performance—is crucial. Additionally, examining differences in segmental body characteristics (e.g., limb length, girths) and their possible relationship with performance in FF is important. At present, standardized reference values specific to FF are lacking. These values could support talent identification, player development, and performance monitoring, as has been observed in other team sports (19, 20).

We recommend that future studies with similar aims consider the limitations and methodological aspects discussed above, in order to adopt more standardized measurement protocols. Furthermore, it is important to involve as many countries as possible to ensure findings are more representative of the global FF population. Another relevant direction for future research would be to explore correlations between anthropometric variables, playing positions, and game performance metrics.

## 5 Limitations

The first limitation was the low and heterogeneous sample size, as not all teams participated in the study. This was due, in part, to the voluntary nature of team participation. However, a statistically significant sample for this type of population was obtained, based on the relevant statistical principles applied. In addition, although FF players from the highest competitive categories were assessed, the sample included only athletes from European and Western Asian countries, excluding players from other continents. Therefore, our results mainly characterize the European FF population. Another limitation is that certain factors—such as players’ rest patterns, dietary conditions or physical performance—were not standardized. It is also important to note that this study used a cross-sectional design, which limits the ability to establish causal relationships between body composition and performance. Despite these limitations, this research represents the first attempt to propose a range of indicative anthropometric values for FF players.

Measurements were not taken under controlled conditions, as it was impossible to schedule all measurement sessions before exercise and was established a wide time range in which the anthropometric assessments were performed due to the logistical constraints inherent in data collection during an international championship with pre-set competition and training schedules. Furthermore, given the characteristics of this championship and the time set by the coach of each team to carry out the anthropometric measurements was limited. This aspect made it difficult to carry out two or three measurements taking into account the technical measurement error (TEM). The standardized ISAK protocol was always followed, and emphasis was placed on the recommendations that athletes should follow to reduce inter-evaluator error, although diurnal variability in hydration, food intake, and previous physical exercise could have influenced the measurements. Also, it could not control the room temperature during data collection. Heat can induce hyperemia due to raised skin temperature and blood flow, which may increase skinfold thickness. These factors could also influence body mass and girth measurements due to transient physiological changes.

One of the strengths of this study is that the researchers who conducted the anthropometric assessments were trained and accredited by the International Society for the Advancement of Kinanthropometry (ISAK) at Level 2 or 3 (28).

This research presents, for the first time, sex-differentiated body composition data of professional FF players. These findings may serve as a useful reference for medical and technical staff when setting and individualizing training objectives to maximize performance. Furthermore, the results offer a solid foundation for developing more personalized and effective training and nutrition programss for FF players, addressing the specific needs and characteristics of each sex.

## 6 Conclusion

This study reveals significant anthropometric and body composition differences between male and female elite flag football (FF) players, contributing to a better understanding of body composition requirements in the sport. Men are generally taller and heavier than women, with notable differences in bone and muscle mass. Although adipose mass does not differ significantly between sexes, skinfold thickness is greater in women. Muscle mass is substantially higher in men, highlighting the importance of sex-specific training programs.

The distribution of adipose tissue also varies by sex, with women showing higher values. Despite greater body mass and height in men, hip and thigh measurements are similar between sexes. Bone lengths and breadths are generally larger in men, which may influence both performance and injury risk.

Somatotype analysis further illustrates the differences in body structure, as reflected in the adipose-muscle and muscle-bone indices. These indicate that men have less adipose mass tissue per kilogram of muscle mass, and more muscle mass per kilogram of bone mass, compared to women. Indices related to bone length and width, such as the crural and acromioiliac indices, also highlight structural differences between sexes, with implications for performance and injury prevention. These aspects should be explored in future research.

This study presents, for the first time, sex-differentiated body composition data for professional FF players. These findings may serve as a reference for medical and technical staff when individualizing training objectives to maximize performance. In addition, the results offer a solid foundation with normative reference values for developing more personalized and effective training and nutrition programs, addressing the specific needs and potential of each sex.

## Data Availability

The original contributions presented in this study are included in this article/supplementary material, further inquiries can be directed to the corresponding author.
